# A Method of Capping Exposed Pulps

**Published:** 1885-02

**Authors:** W. M. Ramsdell

**Affiliations:** Brooklyn, N. Y.


### A METHOD OF CAPPING EXPOSED PULPS.

BY W. M. RAMSDELL, D. D. S., BROOKLYN, N. Y.

In the December (1884) number of the INDEPENDENT PRACTITIONER, "A. B. M." asks some dentist of experience to briefly describe the method he has found most successful in capping exposed pulps.

Inasmuch as this is a far-reaching subject, and one which involves a great variety of circumstances and considerations, I do not wish to be understood as attempting to give (under the above heading) specific rules to be applied to each and every case, as it comes to hand in daily practice. It is by continual modification of principles, and their intelligent adaptation to specific cases, that we reach the highest goal.

In treating exposed pulps, I find in my practice comparatively few cases where the pulp is really exposed to any considerable extent, or where by very careful and exact manipulation lacerating or wounding of the pulp may not be avoided, and it is to this class of cases principally that I apply the following mode of treatment, with which in a trial of several years I have had very general success.

After adjusting the rubber dam and carefully removing with a spoon excavator all extraneous matter, I proceed to dry the cavity as thoroughly as possible; if there is pain I touch lightly with creosote and oil of cloves. Then with an extremely sharp spoon excavator of the desired curve, I proceed very carefully to remove such portion of the super-imposed decalcified dentine as can safely be done without increasing (to any great extent) the exposure, working from the center toward the walls of the cavity, being careful not to "peel up" thick layers of decalcified dentine, but rather to shave off little by little from that portion which I desire to remove from the immediate proximity of the pulp, excavating more thoroughly as I approach the walls of the cavity. After the excavating is completed, I moisten a piece of court plaster, and with the point of a pen-knife remove enough of the plaster to cover the exposure and immediate adjacent parts, and apply while moist.

Now for the cap: I believe that in order to be in the broadest sense effectual, the cap must not only be made of a non-conducting and a non-irritating material, but it must also be so constructed and applied that it will protect the pulp from any pressure whatever, either from its own presence, from the pressure caused by the insertion of the filling material, or from the expansion of the material after its insertion.

A cap which I have found to essentially fill the above requirements may be made and applied in the following manner: take a piece of red cedar, such as we use in connection with pumice-stone for cleansing the teeth, and with a sharp pen-knife reduce one side near the end to a flat surface; then with the engine and a good-sized bur, bur out a saucer-shaped cavity or depression near the end of the stick large enough to cover the exposure and adjacent parts; then with the pen-knife trim it down from the back side until you have formed a miniature saucer, leaving a little neck connecting it with the stick ; just before severing it from the stick, immerse it in a solution of gutta percha and chloroform, of the consistency of cream, giving it a flirt to remove the superfluous solution ; after the chloroform has evaporated sever it from the stick, pick it up on the point of an excavator and put it in place. I sometimes touch the edges of the cap with a solution of Canada balsam and chloroform before carrying it to the cavity ; it then sticks just where you put it. This done, I usually cover the cap and bottom of the cavity with a *thin* layer of Hill's stopping, and fill the remainder of the cavity with oxy-phosphate of zinc.

If, however, I consider the case of doubtful character, or wish to finish the filling with metal, I fill the cavity about half full of the phosphate of zinc and finish with Hill's stopping. I then leave the case until some future time.

These little caps may be filled with medicated paste, and used in treating exposed pulps in various conditions.

Some critics may say that the making of these caps would occupy too much valuable time. I make one in a minute and a half; to coat them with gutta-percha takes a little longer, especially if the solution is thin, and they have to be "dipped" two or three times ; but all delay may be avoided by making them beforehand in assorted sizes.

